# Acute Chemogenetic Activation of CamKIIα-Positive Forebrain Excitatory Neurons Regulates Anxiety-Like Behaviour in Mice

**DOI:** 10.3389/fnbeh.2019.00249

**Published:** 2019-10-29

**Authors:** Sonali S. Salvi, Sthitapranjya Pati, Pratik R. Chaudhari, Praachi Tiwari, Toshali Banerjee, Vidita A. Vaidya

**Affiliations:** Department of Biological Sciences, Tata Institute of Fundamental Research, Mumbai, India

**Keywords:** DREADD, hM3Dq, hM4Di, clozapine-*N*-oxide, anxiety, open field test, light–dark avoidance test, elevated plus maze

## Abstract

Anxiety disorders are amongst the most prevalent mental health disorders. Several lines of evidence have implicated cortical regions such as the medial prefrontal cortex, orbitofrontal cortex, and insular cortex along with the hippocampus in the top–down modulation of anxiety-like behaviour in animal models. Both rodent models of anxiety, as well as treatment with anxiolytic drugs, result in the concomitant activation of multiple forebrain regions. Here, we sought to examine the effects of chemogenetic activation or inhibition of forebrain principal neurons on anxiety and despair-like behaviour. We acutely activated or inhibited Ca^2+^/calmodulin-dependent protein kinase II α (CamKIIα)-positive forebrain excitatory neurons using the hM3Dq or the hM4Di Designer Receptor Exclusively Activated by Designer Drug (DREADD) respectively. Circuit activation was confirmed via an increase in expression of the immediate early gene, c-Fos, within both the hippocampus and the neocortex. We then examined the influence of DREADD-mediated activation of forebrain excitatory neurons on behavioural tests for anxiety and despair-like behaviour. Our results indicate that acute hM3Dq DREADD activation of forebrain excitatory neurons resulted in a significant decline in anxiety-like behaviour on the open field, light–dark avoidance, and the elevated plus maze test. In contrast, hM3Dq DREADD activation of forebrain excitatory neurons did not alter despair-like behaviour on either the tail suspension or forced swim tests. Acute hM4Di DREADD inhibition of CamKIIα-positive forebrain excitatory neurons did not modify either anxiety or despair-like behaviour. Taken together, our results demonstrate that chemogenetic activation of excitatory neurons in the forebrain decreases anxiety-like behaviour in mice.

## Introduction

Multiple cortical circuits including the medial prefrontal cortex (mPFC) (Davidson, 2002; Tovote et al., 2015), orbitofrontal cortex (Milad and Rauch, 2007), anterior insula (Paulus and Stein, 2006), primary motor cortex (Li et al., 2018), somatosensory cortices (Rauch, 1995), and the hippocampal subfields (Shin and Liberzon, 2010; Calhoon and Tye, 2015) are implicated in the modulation of anxiety-like behaviour. These circuits contribute to adaptive emotional control, via the top down-regulation of several components of limbic neurocircuitry that modulate anxiety-like behaviour, such as the amygdala (Adhikari, 2014; Makovac et al., 2016), septum (Parfitt et al., 2017), bed nucleus of the stria terminalis (BNST) (Adhikari, 2014), and hypothalamus (Jimenez et al., 2018). While several previous studies using rodent models have dissected the contribution of individual cortical and subcortical circuits in the modulation of specific anxiety-like behavioural responses, it is likely that diverse brain regions, including multiple cortical circuits, would be concomitantly recruited in an ethological context. This is supported by studies in diverse rodent models associated with increased anxiety-like behaviour, such as immobilisation stress (Ons et al., 2004), air puff (Duncan et al., 1996), swim stress (Molteni et al., 2008), exposure to novel environments such as an open field (Santini et al., 2011), elevated plus maze (EPM) (Muigg et al., 2009), and exposure to cat odour (Úbeda-Contreras et al., 2018), as well as in response to pharmacological agents that modulate anxiety-like behaviour (Hoehn-Saric et al., 2004; Linden et al., 2004; Wise et al., 2007). Such altered neuronal activation patterns across multiple forebrain regions have been observed in these studies as inferred using immunoreactivity for the immediate early genes, c-Fos, egr2, and Arc. This is also supported by clinical observations in humans with high anxiety using both functional magnetic resonance imaging and positron emission tomography indicating the involvement of broad cortical regions, including the frontal cortex (Wang et al., 2016), cingulate cortex (Eser et al., 2009), orbitofrontal cortex (Breiter, 1996), insula (Paulus and Stein, 2006), and hippocampus (Cha et al., 2016).

Anxiolytics, which are pharmacological agents routinely used to treat patients with anxiety disorder, include anxiolytics that target the ion channels, as well as those that act via G-protein coupled receptors (GPCRs) (Griebel and Holmes, 2013). Systemic administration of these compounds also modulates activity in broad telencephalic regions such as the frontal cortex (Wise et al., 2007; Bechtholt et al., 2008), prefrontal cortex (Maslowsky et al., 2010), cingulate cortex (Beck and Fibiger, 1995), and the hippocampus (Beck and Fibiger, 1995; de Medeiros et al., 2005). Spatiotemporal modulation of specific neurocircuits using optogenetics (Tye and Deisseroth, 2012; Nieh et al., 2013) and chemogenetics (Burnett and Krashes, 2016; Whissell et al., 2016) has substantially advanced the understanding of the neurocircuitry that regulates mood-related behaviours. These studies capitalise on topographically restricted neuronal manipulation, and have been instrumental in characterising local microcircuits and neuronal pathways, along with contributions made by specific neuronal cell-types, to explain the regulation of different facets of mood-related behaviours across diverse behavioural paradigms (Tye and Deisseroth, 2012; Burnett and Krashes, 2016). Although these studies have contributed to providing a working understanding of the neurocircuitry of emotional behaviours, the effects of neuronal activity perturbations that recruit broader circuits concomitantly such as within the forebrain, are still not well-understood.

In this study, we sought to investigate the effects of altered activity within the broad forebrain circuits including the neocortex and hippocampus on anxiety and despair-like behaviour using chemogenetics. Excitatory neurons constitute a majority of the neuronal subpopulations within the forebrain (Megìas et al., 2001; Douglas and Martin, 2004). We used transgenic mouse lines expressing engineered human muscarinic receptors, i.e., the excitatory hM3Dq or the inhibitory hM4Di Designer Receptors Exclusively Activated by Designer Drugs (DREADDs) (Armbruster et al., 2007; Nawaratne et al., 2008), in Ca^2+^/calmodulin-dependent protein kinase II α (CamKIIα)-positive excitatory neurons of the forebrain, to examine effects of forebrain principal neuron activation and inhibition on mood-related behaviours. Our results indicate that acute DREADD ligand [clozapine-*N*-oxide (CNO)] mediated hM3Dq activation of CamKIIα-positive forebrain excitatory neurons evokes anxiolysis in the open field test, light–dark avoidance test and EPM test, but does not influence despair-like behaviour. Acute CNO-mediated hM4Di DREADD inhibition of CamKIIα-positive forebrain excitatory neurons did not influence either anxiety or despair-like behaviour. These findings indicate that acute chemogenetic activation of forebrain excitatory neurons exerts anxiolytic effects across diverse anxiety-related behavioural tasks.

## Materials and Methods

### Animals

Bigenic CamKIIα-tTA:TetO-hM3Dq, CamKIIα-tTA:TetO-hM4Di, and C57BL/6J mice were bred in the Tata Institute of Fundamental Research (TIFR) animal facility and were maintained on a 12-h light–dark cycle (lights on from 7:00 AM) with *ad libitum* access to food and water. CamKIIα-tTA transgenic mice were a kind gift from Dr. Christopher Pittenger, Yale School of Medicine. TetO-hM3Dq [Cat. No. 014093; Tg(tetO-CHRM3^∗^)1Blr/J], TetO-hM4Di [Cat No. 024114; Tg(tetO-CHRM4^∗^)2Blr/J] mouse lines, and C57BL/6J mice were purchased from Jackson Laboratories, United States. All bigenic mouse lines were maintained on a doxycycline–free diet from development onwards to facilitate transgene expression. Genotypes were determined using PCR based analysis. All experimental procedures were carried out following the guidelines of the Committee for the Purpose of Control and Supervision of Experiments on Animals (CPCSEA), Government of India and were approved by the TIFR institutional animal ethics committee.

### Experimental Paradigm

Age-matched (3–5 months), adult male bigenic CamKIIα-tTA:TetO-hM3Dq (*n* = 9–10/group), CamKIIα-tTA:TetO-hM4Di (*n* = 9–10/group) mice, and C57BL/6J (*n* = 10–12/group) mice were intraperitoneally injected with either 0.5 mg/kg CNO (Cat no. 4936; Tocris, United Kingdom) or vehicle (0.9% NaCl). Behavioural analysis on the open field test (OFT), elevated plus maze test (EPM), light–dark (LD) avoidance test, tail suspension test (TST), and forced swim test (FST) were performed 30 min following CNO/vehicle injection. An interim washout period (7–10 days) was provided between all behavioural tests, with the tests for anxiety-like behaviours preceding the despair-like behaviour tests. A separate cohort of adult CamKIIα-tTA:TetO-hM3Dq (*n* = 4/group) and CamKIIα-tTA:TetO-hM4Di (*n* = 4/group) male mice were sacrificed 2 h following 0.5 mg/kg CNO or vehicle administration for western blotting experiments. An additional cohort of CamKIIα-tTA:TetO-hM3Dq (*n* = 9–10/group) adult male mice (5 months) were injected with either 0.5 mg/kg CNO or vehicle, and sacrificed 2 h later by transcardial perfusion with 4% paraformaldehyde for immunohistochemical analysis.

### Behavioural Assays

To assess anxiety-like behaviour, vehicle and CNO-treated CamKIIα-tTA:TetO-hM3Dq, vehicle and CNO-treated CamKIIα-tTA:TetO-hM4Di, and vehicle and CNO-treated C57BL/6J male mice were subjected to OFT, EPM test, and LD avoidance tests. Despair-like behaviour was assessed using the TST, followed by FST. Care was taken to clean and dry the behavioural arenas with ethanol between individual trials during each behavioural test. Behavioural tests on the same cohort of mice were set apart by an interim washout period of 7–10 days. Behavioural analysis on the OFT and EPM tests were performed using Ethovision XT 11 (Noldus, Netherlands). Behavioural analysis on the LD avoidance test, TST, and FST were carried out manually from video recordings, by an experimenter blind to the treatment groups.

#### Open Field Test

Mice were introduced at random into a corner of the OFT box (40 cm × 40 cm × 40 cm) and allowed to explore the arena for a duration of 5 min. Behaviour was recorded using an infrared overhead camera (Harvard Apparatus, United States) followed by analysing anxiety-like behaviour, using the measures of total distance travelled, percent distance travelled in centre and percent time spent in the centre of the arena (20 cm × 20 cm), as well as number of entries made to the centre of the arena.

#### Elevated Plus Maze Test

The EPM test consisted of an elevated platform raised 50 cm above the ground with opposing open and closed arms. Mice were introduced into the centre of the EPM, facing the open arms and allowed to explore for a total duration of 10 min. Ethovision analysis for anxiety-like behaviour was based on assessing measures of total distance travelled, percent distance travelled in the open arms, percent time spent in the open arms, and number of entries to the open arms of the EPM.

#### LD Avoidance Test

Mice were introduced into a box with a light and a dark chamber connected through an entrance and allowed to explore the arena for 10 min. Video recordings were then manually analysed for percent time spent, and number of entries into the light chamber to assess anxiety-like behaviour.

#### Tail Suspension Test

Tail suspension test was performed by suspending the mice by their tails 50 cm from the ground and behaviour was recorded for a duration of 6 min. Despair-like behaviour was assessed based on percent time spent immobile by the mice during the last 5 min of the TST.

#### Forced Swim Test

Forced swim test was performed by introducing mice into a cylindrical Plexiglas chamber filled with water (22°C) and behaviour was recorded for 6 min. Percent time spent immobile by the mice during the last 5 min of the behaviour was used as a measure to assess despair-like behaviour.

### Immunohistochemistry

Vehicle or CNO-treated CamKIIα-tTA:TetO-hM3Dq adult male mice, naive for behavioural testing, were sacrificed by transcardial perfusion with 4% paraformaldehyde 2 h post-drug treatments, for c-Fos immunohistochemistry (IHC). Free-floating (40 μM) coronal sections cut on the vibratome (Leica, Germany) were incubated with the blocking solution (10% horse serum, 0.3% TritonX-100 in 0.1M Phosphate buffer) at room temperature for 2 h. Sections were then incubated with rabbit anti-c-Fos antibody (1:1000, Cat no. 2250, Cell Signalling Technology, United States) for 2 days at 4°C, followed by sequential washes, and incubation with the secondary antibody (biotinylated goat anti-rabbit, 1:500, Cat no. BA9400, Vector Labs, United States) for 2 h at room temperature. Signal amplification was performed using an Avidin-biotin complex based system (Vector lab, Vectastain ABC kit Elite PK1600, United States) and visualised using the substrate, Diaminobenzidine tetrahydrochloride (Cat no. D5905, Sigma-Aldrich, United States).

### Cell Counting Analysis

Quantitative analysis of c-Fos immunopositive cells was performed in specific hippocampal subfields namely the CA1, CA3, and dentate gyrus (DG) using a brightfield microscope (Zeiss Axioskop 2 plus, Germany) at a magnification of 200X. Six sections of each region, spanning the rostrocaudal extent of the hippocampus at a periodicity of 200 μm, were selected per animal. Cell counting analysis was carried out by an experimenter blind to the treatment groups. Contours were drawn for each hippocampal subfield using Neurolucida explorer (MBF Biosciences, United States) and an average area in mm^2^ for the CA1, CA3, and DG hippocampal subfields was calculated for both vehicle-treated controls and CNO treated animals. Results are expressed as the number of c-Fos-positive cells per mm^2^ for each hippocampal subfield analysed.

### Western Blotting

Western blotting analysis was performed on tissue samples that were snap-frozen in liquid nitrogen. Tissue homogenisation was carried out in Radioimmunoprecipitation assay (RIPA) buffer (10 mM Tris-Cl (pH 8.0), 1 mM EDTA, 0.5 mM EGTA, 1% Triton X-100, 0.1% sodium deoxycholate, 0.1% SDS, 140 mM NaCl) using a Dounce homogeniser. Protease and phosphatase inhibitors (Sigma-Aldrich, United States) were added to the buffer prior to lysis. Estimation of the protein concentration was performed using a Quantipro BCA assay kit (Sigma-Alrich, United States). Protein lysates (50 μg) were resolved using a 10% sodium dodecyl sulphate polyacrylamide gel electrophoresis (SDS PAGE) system and transferred onto polyvinylidene fluoride membranes. Blots were then incubated with blocking solution (5% milk in TBST) and exposed to either rabbit anti-HA (1:1500, Cat no. H6908, Sigma-Aldrich, United States), rabbit anti-c-Fos (1:1000, Cat no. 2250, Cell Signalling Technology, United States), or rabbit anti-actin (1: 10,000, Cat no. AC026, Abclonal Technology, United States) primary antibodies made in 5% milk. Subsequent to sequential washes, blots were incubated with HRP conjugated goat anti-rabbit (1:6000, Cat no. AS014, Abclonal Technology, United States) secondary antibody and the signal was visualised with the GE Amersham Imager 600 (GE life sciences, United States) using a western blotting detection kit (WesternBright ECL, Advansta, United States). Densitometric analysis of the blots was performed using ImageJ software.

### Statistical Analysis

Statistical analyses was performed using the two-tailed unpaired Student’s *t*-test (Instat, GraphPad Software, Inc., United States) with significance determined at a *p*-value < 0.05. Results were subjected to the Kolmogorov–Smirnov test to determine normality prior to statistical analysis. Results are expressed as mean ± standard error of mean (SEM).

## Results

### hM3Dq and hM4Di DREADD-Mediated Activation and Inhibition of CamKIIα-Positive Forebrain Excitatory Neurons

**FIGURE 1:**
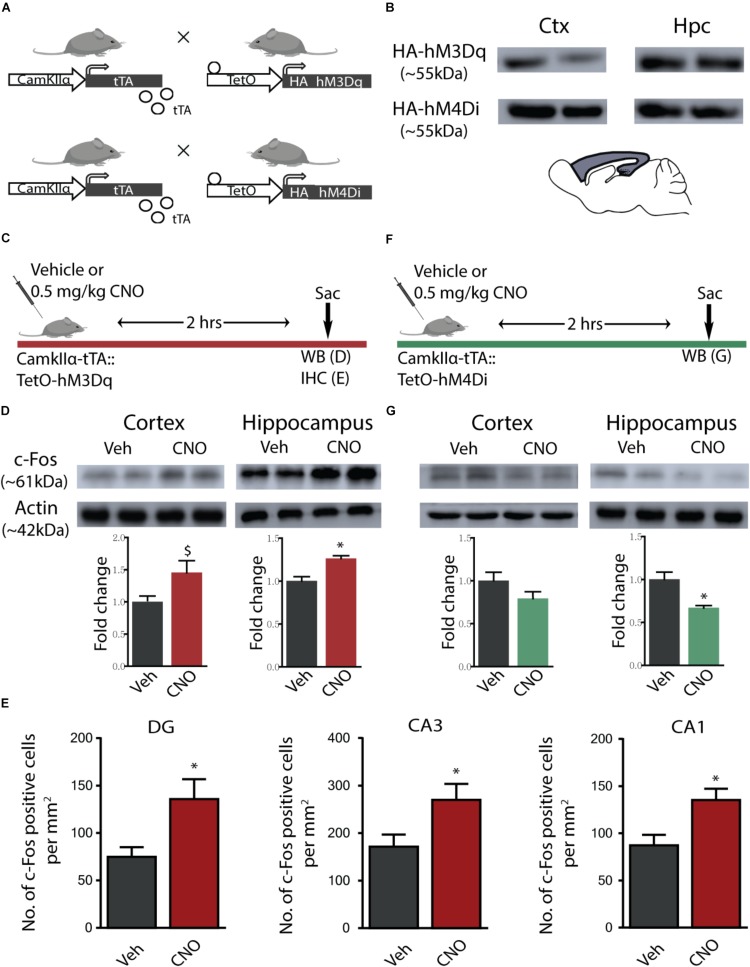
hM3Dq and hM4Di DREADD-mediated activation and inhibition of CamKIIα-positive forebrain excitatory neurons. **(A)** Shown is a schematic of the genetic strategy used to create bigenic CamKIIα-tTA:TetO-hM3Dq and CamKIIα-tTA:TetO-hM4Di mice to drive expression of the hM3Dq and hM4Di DREADD in forebrain CamKIIα-positive excitatory neurons respectively. **(B)** Shown is protein expression of the HA antigen in the forebrain circuits of the cortex (Ctx) and the hippocampus (Hpc) confirming expression of the HA-tagged hM3Dq (HA-hM3Dq) and hM4Di (HA-hM4Di) DREADD. **(C)** Shown is a schematic of the experimental paradigm used to confirm the activation of the hM3Dq DREADD in the forebrain. **(D)** Western blotting analysis revealed that adult bigenic CamKIIα-tTA:TetO-hM3Dq mice injected with CNO (0.5 mg/kg) showed significant induction in c-Fos protein expression in the hippocampus (*n* = 4/group), and a trend toward an increase in the cortex. **(E)** Immunohistochemical analysis indicated that acute CNO-mediated hM3Dq DREADD activation resulted in a significant increase in the number of c-Fos positive cells in the hippocampal subfields, namely the Dentate gyrus (DG), CA3, and CA1 (*n* = 9–10/group). **(F)** Shown is a schematic of the experimental paradigm used to confirm the activation of the hM4Di DREADD in the forebrain. **(G)** Western blotting analysis revealed that adult bigenic CamKIIα-tTA:TetO-hM4Di mice injected with CNO (0.5 mg/kg) showed a significant decline in c-Fos protein expression in the hippocampus (*n* = 4/group), and no change in c-Fos protein levels in the cortex. Results are expressed as the mean ± SEM, ^∗^*p* < 0.05, ^$^*p* = 0.07, as compared to vehicle-treated control mice using the two-tailed, unpaired Student’s *t*-test.

Bigenic CamKIIα-tTA:TetO-hM3Dq and CamKIIα-tTA:TetO-hM4Di mice were generated for the chemogenetic manipulation of CamKIIα-positive forebrain excitatory neurons (Figure 1A). Expression of the HA-tagged excitatory (hM3Dq) and inhibitory (hM4Di) DREADDs in the forebrain was confirmed using western blotting for the HA antigen in the cortex and hippocampus (Figure 1B). The excitatory and inhibitory DREADD expression was not observed within hindbrain regions such as the cerebellum (data not shown).

We investigated the hM3Dq DREADD-mediated activation of the cortex and hippocampus, 2 h following acute CNO (0.5 mg/kg) administration using western blotting and immunohistochemistry for the neuronal activity marker, c-Fos (Figure 1C). Western blotting analysis clearly indicated a significant increase in expression of c-Fos protein following acute CNO treatment in the hippocampi (*p* = 0.004) derived from bigenic CamKIIα-tTA:TetO-hM3Dq, as compared to their vehicle-treated controls (Figure 1D), and a trend (*p* = 0.07) toward a significant induction of c-Fos protein levels in the cortex (Figure 1D). Additionally, immunohistochemical analysis indicated a significant increase in c-Fos positive cell numbers within the dentate gyrus (DG), CA1, and CA3 hippocampal subfields of CNO treated CamKIIα-tTA:TetO-hM3Dq mice (Figure 1E, *p* = 0.015 DG, *p* = 0.009 CA1, *p* = 0.03 CA3). We next examined the influence of hM4Di DREADD-mediated inhibition of the cortex and hippocampus 2 h following acute CNO (0.5 mg/kg) administration using western blotting for the neuronal activity marker, c-Fos (Figure 1F). Western blotting analysis revealed a significant decline in the c-Fos protein in the hippocampus (*p* = 0.01) and no significant change noted for c-Fos protein levels in the cortex, following acute CNO-mediated hM4Di DREADD stimulation (Figure 1G).

### Acute CNO-Mediated DREADD Activation of CamKIIα-Positive Forebrain Excitatory Neurons Reduces Anxiety-Like Behaviour on the OFT

**FIGURE 2:**
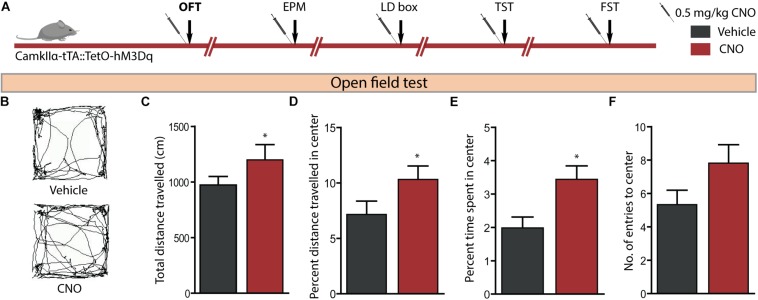
Acute CNO-mediated DREADD activation of CamKIIα-positive forebrain excitatory neurons reduces anxiety-like behaviour on the open field test (OFT). **(A)** Shown is a schematic of the experimental paradigm used to determine the influence of acute CNO (0.5 mg/kg)-mediated hM3Dq activation of CamKIIα-positive forebrain excitatory neurons in adult male bigenic CamKIIα-tTA:TetO-hM3Dq mice on anxiety and despair-like behaviour. Treatment groups were subjected to a battery of anxiety and despair-like behavioural tasks, with an interim washout period of 7–10 days (*n* = 9–10/group). Shown in this figure is the anxiety-like behaviour on the OFT **(B–F)** following acute hM3Dq DREADD activation of forebrain excitatory neurons, commencing 30 min following CNO administration. **(B)** Shown are representative tracks from a vehicle and CNO treated CamKIIα-tTA:TetO-hM3Dq mouse in the OFT. CNO-mediated hM3Dq DREADD activation of CamKIIα-positive forebrain excitatory neurons resulted in decline in anxiety-like behaviour on the OFT as revealed by significant increases in the total distance travelled **(C)**, percent distance travelled in the centre **(D)**, and percent time spent in the centre **(E)** of the OFT arena as compared to the vehicle-treated group. No significant difference was observed in the number of entries made to the centre of the arena **(F)**. Results are expressed as the mean ± SEM (*n* = 9–10/group), ^∗^*p* < 0.05, as compared to vehicle-treated CamKIIα-tTA:TetO-hM3Dq mice, two-tailed, unpaired Student’s *t*-test. EPM, elevated plus maze test; LD, light–dark avoidance test; TST, tail suspension test; FST, forced swim test.

We then examined the influence of acute CNO (0.5 mg/kg)-mediated hM3Dq DREADD activation of CamKIIα-positive forebrain excitatory neurons on a battery of anxiety and despair-like behavioural tasks. Behavioural testing on the OFT was performed 30 min following acute CNO administration (Figure 2A). Acute hM3Dq DREADD-mediated activation of forebrain excitatory neurons evoked a significant decline in anxiety-like behaviour on the OFT (Figures 2B–F). This was indicated by an increase in the total distance travelled (Figure 2C, *p* = 0.006), percent distance travelled within the centre (Figure 2D, *p* = 0.006) and percent time spent within the centre (Figure 2E, *p* = 0.0003) of the OFT arena by CNO-treated CamKIIα-tTA:TetO-hM3Dq mice as compared to their vehicle-treated controls. The number of entries made to the centre of the OFT arena (Figure 2F) did not differ across the groups. These results indicate an anxiolytic response in the OFT following acute CNO-mediated hM3Dq DREADD activation of the forebrain excitatory neurons.

### Acute CNO-Mediated DREADD Activation of CamKIIα-Positive Forebrain Excitatory Neurons Reduces Anxiety-Like Behaviour on the Light–Dark Avoidance and Elevated Plus Maze Test

**FIGURE 3:**
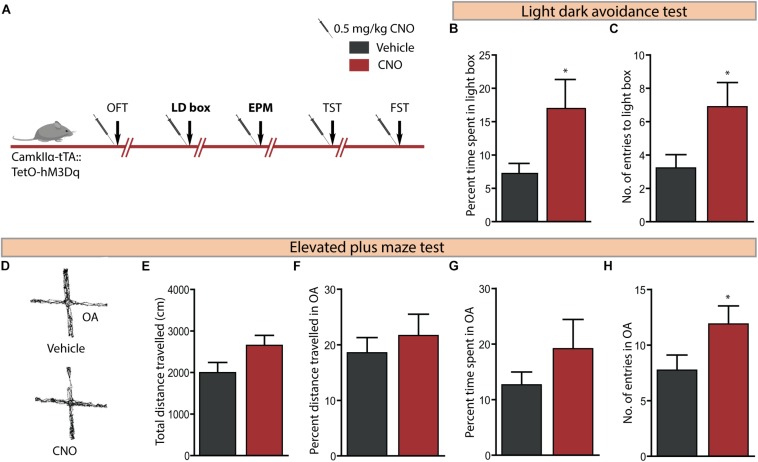
Acute CNO-mediated DREADD activation of CamKIIα-positive forebrain excitatory neurons reduces anxiety-like behaviour on the light dark avoidance and EPM test. **(A)** Shown is a schematic of the experimental paradigm used to determine the influence of acute CNO (0.5 mg/kg)-mediated hM3Dq activation of CamKIIα-positive forebrain excitatory neurons in adult male bigenic CamKIIα-tTA:TetO-hM3Dq mice on anxiety and despair-like behaviour. Treatment groups were subjected to a battery of anxiety and despair-like behavioural tasks, with an interim washout period of 7–10 days (*n* = 9–10/group). Shown in this figure is the anxiety-like behaviour on the light–dark avoidance test (LD) **(B,C)** and EPM test **(D–H)** following acute hM3Dq DREADD activation of forebrain excitatory neurons, commencing 30 min following CNO administration. CNO-mediated hM3Dq DREADD activation of CamKIIα-positive forebrain excitatory neurons resulted in a decline in anxiety-like behaviour on the LD avoidance test as revealed by significant increases in percent time spent in the light chamber **(B)** and number of entries made to the light chamber **(C)** of the LD avoidance test arena. **(D)** Shown are representative tracks from a vehicle and CNO treated CamKIIα-tTA:TetO-hM3Dq mouse in the EPM test. CNO-mediated hM3Dq DREADD activation of CamKIIα-positive forebrain excitatory neurons resulted in no change in the total distance travelled **(E)**, percent distance travelled in the open arms (OA) **(F)**, and percent time spent in the open arms **(G)** of the EPM compared to the vehicle-treated group. A significant increase in the number of entries to the open arms of the EPM arena **(H)** was noted following hM3Dq DREADD activation of forebrain excitatory neurons. Results are expressed as the mean ± SEM (*n* = 9–10/group), ^∗^*p* < 0.05, as compared to vehicle-treated CamKIIα-tTA:TetO-hM3Dq mice, two-tailed, unpaired Student’s *t*-test. OFT, open field test; TST, tail suspension test; FST, forced swim test.

We then assessed the effect on anxiety-like behaviour following hM3Dq DREADD-mediated activation of CamKIIα-positive forebrain excitatory neurons using the LD avoidance and EPM test, with an interim washout period of 7–10 days between behavioural tasks (Figure 3A). Behavioural analysis on the LD avoidance test revealed a decline in anxiety-like behaviour (Figures 3B,C), as noted with a significant increase in both the percent time spent in the light chamber (Figure 3B, *p* = 0.05) and number of entries to the light chamber (Figure 3C, *p* = 0.04) by the CNO-treated cohort. We next assessed the influence of hM3Dq DREADD activation of forebrain excitatory neurons on anxiety-like behaviour on the EPM test (Figures 3D–H). We noted a significant increase in the number of entries made into the open arms (Figure 3H, *p* = 0.02) of the EPM. We did not observe any significant difference on other measures, namely the total distance travelled in the EPM arena (Figure 3E), percent distance travelled in the open arms (Figure 3F), and percent time spent in the open arms (Figure 3G) following CNO-mediated hM3Dq DREADD activation. These results indicate that acute CNO-mediated hM3Dq DREADD activation in the forebrain excitatory neurons evokes a prominent anxiolytic response on the LD avoidance test and on specific measures of the EPM test.

### Acute CNO-Mediated DREADD Activation of CamKIIα-Positive Forebrain Excitatory Neurons Does Not Influence Despair-Like Behaviour

**FIGURE 4:**
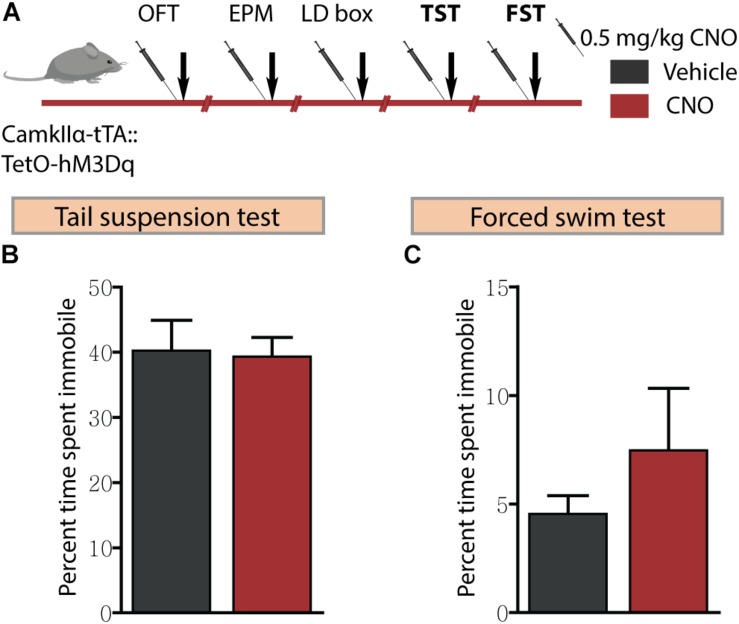
Acute CNO-mediated DREADD activation of CamKIIα-positive forebrain excitatory neurons does not influence despair-like behaviour. **(A)** Shown is a schematic of the experimental paradigm used to determine the influence of acute CNO (0.5 mg/kg)-mediated hM3Dq activation of CamKIIα-positive forebrain excitatory neurons in adult male bigenic CamKIIα-tTA:TetO-hM3Dq mice on despair-like behaviour. Treatment groups were subjected to the TST and the FST to assess despair-like behaviour, with an interim washout period of 7–10 days (*n* = 9–10/group). CNO-mediated hM3Dq DREADD activation of CamKIIα-positive forebrain excitatory neurons did not alter despair-like behaviour on either the TST or FST, with no difference noted in percent time spent immobile in the TST **(B)** and the FST **(C)** tests, as compared to vehicle-treated CamKIIα-tTA:TetO-hM3Dq mice. Results are expressed as the mean ± SEM (*n* = 9–10/group), two-tailed, unpaired Student’s *t*-test.

Given we noted robust anxiolytic responses on multiple behavioural tests for anxiety-like behaviour following acute hM3Dq DREADD-mediated activation of forebrain excitatory neurons, we next assessed for effects on despair-like behaviour using the TST and FST (Figure 4A). hM3Dq DREADD-mediated activation of forebrain excitatory neurons did not influence despair-like behaviour on either the TST (Figure 4B) or the FST (Figure 4C) with no change noted in percent immobility time in both tasks. These results indicate that acute DREADD-mediated activation of the CamKIIα-positive forebrain excitatory neurons influences anxiety but not despair-like behaviour.

### Acute hM4Di DREADD-Mediated Inhibition of CamKIIα-Positive Forebrain Excitatory Neurons Does Not Influence Anxiety or Despair-Like Behaviour

**FIGURE 5:**
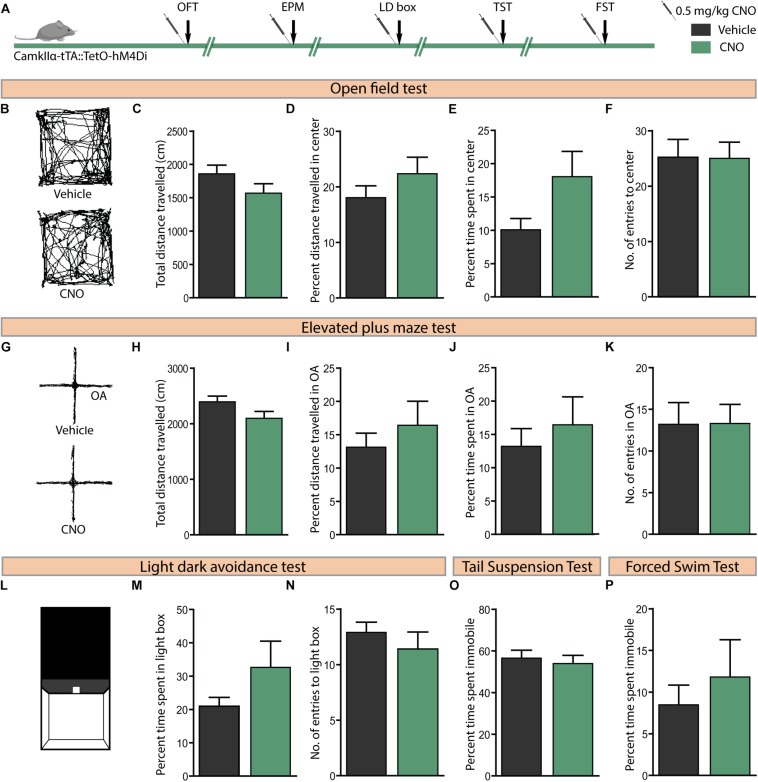
Acute CNO-mediated DREADD inhibition of CamKIIα-positive forebrain excitatory neurons does not alter anxiety or despair-like behaviour. **(A)** Shown is a schematic of the experimental paradigm used to determine the influence of acute CNO (0.5 mg/kg)-mediated hM4Di DREADD inhibition of CamKIIα-positive forebrain excitatory neurons in adult male bigenic CamKIIα-tTA:TetO-hM4Di mice on anxiety and despair-like behaviour. Treatment groups were subjected to a battery of anxiety and despair-like behavioural tasks, with an interim washout period of 7–10 days (*n* = 9–10/group). **(B)** Shown are representative tracks from a vehicle and CNO-treated CamKIIα-tTA:TetO-hM4Di mouse in the OFT **(B–F)**. CNO-mediated hM4Di DREADD inhibition of CamKIIα-positive forebrain excitatory neurons did not influence anxiety-like behaviour in the OFT as indicated by no change observed in the total distance travelled **(C)**, percent distance travelled in the centre **(D)**, percent time spent in the centre **(E)**, and number of entries made to the centre **(F)** as compared to vehicle-treated controls. **(G)** Shown are representative tracks from a vehicle and CNO treated CamKIIα-tTA:TetO-hM4Di mouse in the EPM test **(G–K)**. CNO-mediated hM4Di DREADD inhibition of CamKIIα-positive forebrain excitatory neurons did not influence the total distance travelled **(H)**, percent distance travelled in the open arms (OA) **(I)**, percent time spent in the open arms **(J)**, and number of entries to the open arms **(K)** of the EPM. **(L)** Shown is an illustration of the chamber used in the light–dark (LD) avoidance test **(L–N)**. CNO-mediated hM4Di DREADD inhibition of CamKIIα-positive forebrain excitatory neurons did not influence percent time spent in the light chamber **(M)** and number of entries made to the light chamber **(N)**. CNO-mediated hM4Di DREADD inhibition of CamKIIα-positive forebrain excitatory neurons did not influence despair-like behaviour in the TST and FST, with no change noted in percent immobility time in both the TST **(O)** and the FST **(P)**, as compared to vehicle-treated CamKIIα-tTA:TetO-hM4Di mice. Results are expressed as the mean ± SEM (*n* = 9–10/group), two-tailed, unpaired Student’s *t*-test.

We then sought to examine the effects of acute CNO (0.5 mg/kg)-mediated hM4Di DREADD inhibition of CamKIIα-positive forebrain excitatory neurons on anxiety-like behaviour in the OFT, EPM, and LD avoidance test and on despair-like behaviour in the TST and FST paradigms (Figure 5A). Acute hM4Di DREADD-mediated inhibition of forebrain excitatory neurons revealed no influence on anxiety-like behaviour in the OFT (Figures 5B–F). This was indicated by no difference on measures of the total distance travelled in the arena (Figure 5C), percent distance travelled in the centre (Figure 5D), percent time spent in the centre (Figure 5E), and number of entries made to the centre of the OFT arena (Figure 5F) between treatment groups. Acute hM4Di DREADD-mediated inhibition of forebrain excitatory neurons did not alter anxiety-like behaviour on the EPM test (Figures 5G–K). No significant differences were noted on measures of the total distance travelled (Figure 5H), percent distance travelled in the open arms (Figure 5I), percent time spent in the open arms (Figure 5J), and number of entries made to the open arms (Figure 5K) between the CNO and vehicle-treated groups. We also assessed anxiety-like behaviour on the light–dark avoidance test (Figures 5L–N), with no difference observed in either percent time spent in the light chamber (Figure 5M) or number of entries made to the light chamber (Figure 5N) following CNO-mediated hM4Di DREADD inhibition of forebrain excitatory neurons. Further, we examined despair-like behaviour on the TST and FST, and noted that percent immobility time was unchanged in both the TST (Figure 5O) and FST (Figure 5P) following hM4Di DREADD-mediated inhibition of forebrain excitatory neurons. Taken together, these results indicate that acute hM4Di DREADD-mediated inhibition of the forebrain excitatory neurons does not influence anxiety-like behaviour on the OFT, EPM, and LD avoidance test and despair-like behaviour on the TST and FST.

### Acute Administration of CNO in C57BL/6J Mice Does Not Influence Anxiety-Like or Despair-Like Behaviour

**FIGURE 6:**
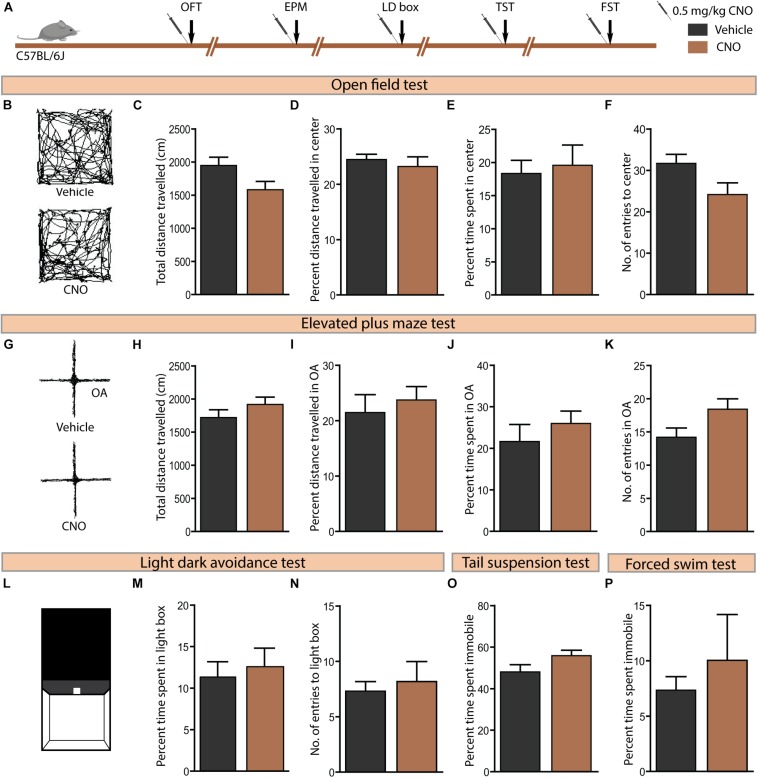
Acute CNO administration in C57BL/6J mice does not influence anxiety or despair-like behaviour. **(A)** Shown is a schematic of the experimental paradigm used to determine the influence of acute CNO (0.5 mg/kg) administration in C57BL/6J mice, the background strain for the bigenic CamKIIα-tTA:TetO-hM3Dq and CamKIIα-tTA:TetO-hM4Di mouse lines, on anxiety and despair-like behaviours. Treatment groups were subjected to a battery of anxiety and despair-like behavioural tasks, with an interim washout period of 7–10 days (*n* = 10–12/group). **(B)** Shown are representative tracks from a vehicle and CNO treated C57BL/6J mouse in the OFT **(B–F)**. CNO administration to C57BL/6J mice did not influence anxiety-like behaviour in the OFT as indicated by no change observed in the total distance travelled **(C)**, percent distance travelled in the centre **(D)**, percent time spent in the centre **(E)**, and number of entries made to the centre **(F)** as compared to vehicle-treated controls. **(G)** Shown are representative tracks from a vehicle and CNO treated C57BL/6J mouse in the EPM test **(G–K)**. CNO administration to C57BL/6J mice did not influence the total distance travelled **(H)**, percent distance travelled in the open arms (OA) **(I)**, percent time spent in the open arms **(J)**, and number of entries to the open arms **(K)** of the EPM. **(L)** Shown is an illustration of the chamber used in the light–dark (LD) avoidance test **(L–N)**. CNO administration to C57BL/6J mice did not influence percent time spent in the light chamber **(M)** and number of entries made to the light chamber **(N)**. CNO administration to C57BL/6J mice did not influence despair-like behaviour in the TST and FST, with no change noted in percent time spent immobile in both the TST **(O)** and the FST **(P)**, as compared to vehicle-treated CamKIIα-tTA:TetO-hM4Di mice. Results are expressed as the mean ± SEM (*n* = 10–12/group), two-tailed, unpaired Student’s *t*-test.

Given concerns that CNO metabolism results in the formation of active metabolites, namely clozapine and *N*-desmethylclozapine (Manvich et al., 2018), which are known to bind to endogenous receptors (Meltzer, 1994; Lameh et al., 2007), it is important to control for potential off-target behavioural effects of CNO administration. We examined the influence of CNO (0.5 mg/kg) administration in C57BL/6J mice, the background strain for the bigenic CamKIIα-tTA:TetO-hM3Dq and CamKIIα-tTA:TetO-hM4Di mouse lines, on anxiety and despair-like behaviours (Figure 6A). Administration of CNO to C57BL/6J mice did not influence anxiety-like behaviour on the OFT (Figures 6B–F) as indicated by no change in the total distance travelled (Figure 6C), percent distance travelled in the centre (Figure 6D), percent time spent in the centre (Figure 6E), and number of entries made to the centre (Figure 6F) of the OFT arena. Similarly, CNO administration to C57BL/6J mice did not influence anxiety-like behaviour on either the EPM (Figures 6G–K) or the LD avoidance test (Figures 6L–N). Next, we assessed despair-like behaviour on the TST and FST. CNO-treated mice showed no significant difference in percent immobility time on either the TST (Figure 6O) or FST (Figure 6P). Taken together, these results provide an important control indicating that acute administration of CNO in C57BL/6J mice, or the generation of CNO metabolites at this dose (0.5 mg/kg, CNO) do not influence anxiety or despair-like behaviour. Thesed control experiments strengthen our observation that the anxiolytic behavioural responses following CNO-mediated hM3Dq DREADD activation of forebrain principal neurons do not involve any off-target actions of CNO, or its metabolites.

## Discussion

Our findings indicate that acute hM3Dq DREADD-mediated activation of CamKIIα-positive forebrain excitatory neurons evokes a significant decline in anxiety-like behaviour on multiple tasks, namely the OFT, LD avoidance, and the EPM test, with no change noted in despair-like behaviour. In contrast, acute hM4Di DREADD-mediated inhibition of CamKIIα-positive forebrain excitatory neurons does not influence anxiety or despair-like behaviour. These observations indicate that acute chemogenetic activation of forebrain excitatory neurons can elicit an anxiolytic behavioural response.

Acute hM3Dq DREADD-mediated activation of forebrain excitatory neurons significantly increased the expression of the immediate early gene, c-Fos, as characterised by western blotting and immunohistochemistry, in the cortex and hippocampus. This indicates that acute CNO administration to CamKIIα-tTA:TetO-hM3Dq mice results in broad circuit activation, within both cortical and hippocampal regions, as expected based on the genetic driver (CamKIIα-tTA) used (Mayford et al., 1996). The nature of c-Fos activation in our study is reflective of the pattern of CamKIIα-tTA driver expression, which has been previously characterised to drive the strongest transgene expression within the hippocampus, in particular the CA subfields, with strong expression also noted in cortical regions (Mayford et al., 1996; Alexander et al., 2009). Prior reports have carried out detailed electrophysiological investigation using the same bigenic mouse line, CamKIIα-tTA:TetO-hM3Dq, used in our study, indicating CNO-evoked depolarisation and enhanced firing rate, through a Gq-coupled GPCR signalling driven mechanism (Alexander et al., 2009). It is important to note that we cannot preclude the possibility that CNO-mediated hM3Dq DREADD stimulation may evoke a differential degree of activation across specific forebrain circuits (Alexander et al., 2009), which should be taken into account in the interpretation of our behavioural results. Nevertheless, such broad activation of multiple forebrain regions is common in diverse ethological contexts (Duncan et al., 1996; Ons et al., 2004; Park and Chung, 2019), as well as in response to therapeutic modalities (Linden et al., 2004; Wise et al., 2007; Bechtholt et al., 2008) used to target mood-related disorders.

To address the influence of acute hM3Dq DREADD-mediated activation of CamKIIα-positive forebrain excitatory neuron on mood-related behaviour, we subjected the mice to a battery of behavioural tasks including OFT, LD avoidance, EPM, TST, and FST. On tasks that assessed avoidance and defencive behaviour based responses namely the OFT, LD avoidance, and EPM, CNO-mediated activation of hM3Dq DREADD resulted in decreased anxiety-like behaviour that was most apparent on the OFT and LD avoidance test on multiple measures. The effects on anxiety-like behaviour in the EPM were restricted to only the measure of the number of entries made to the open arms. Taken together, acute chemogenetic activation of forebrain excitatory neurons clearly results in a reduction in anxiety-like behaviour, but to a different extent based on the behavioural test employed. These results suggest possible task-specific anxiolytic responses that vary in magnitude following hM3Dq DREADD-mediated forebrain excitatory neuron activation. CNO-treated CamKIIα-tTA:TetO-hM3Dq mice also exhibited increased total movement in the OFT, which is in agreement with a prior report of enhanced ambulation following hM3Dq DREADD activation of the CamKIIα-positive excitatory neurons (Alexander et al., 2009). Though the CamKIIα-tTA:TetO-hM3Dq bigenic mouse line has been previously assessed for effects in the EPM, the authors did not report any effect on anxiety-like behaviour (Alexander et al., 2009). Our results also indicate that the most robust anxiolytic effects of DREADD activation in the CamKIIα-tTA:TetO-hM3Dq mouse line are noted in the OFT and LD avoidance test, with a subtle change noted in the EPM. Differential recruitment of specific forebrain circuits following CNO-mediated hM3Dq DREADD activation could contribute to the differences noted in the degree of behavioural responses across tasks such as the OFT, LD avoidance and EPM. While chemogenetic activation of forebrain excitatory neurons reduced anxiety-like behaviour, we did not observe any influence on despair-related behavioural measures in either the TST or FST. Overall, analysis across diverse mood-related behavioural tasks reveal that hM3Dq DREADD activation of CamKIIα-positive excitatory neurons enhances anxiolytic behaviours, with no influence on despair-like responses.

Our studies using bigenic CamKIIα-tTA:TetO-hM4Di mice indicated no effects of CNO treatment on anxiety or despair-like behaviours. Acute CNO administration to CamKIIα-tTA:TetO-hM4Di mice reduced c-Fos expression significantly within the hippocampus, but not in the cortex, with this decline in IEG expression interpreted as indicative of a decline in neuronal activity. This suggests that while the enhancement of Gq-coupled GPCR signalling via hM3Dq DREADD activation in forebrain excitatory neurons is capable of reducing anxiety-like behaviour, acute increases in Gi-coupled GPCR signalling via hM4Di DREADD activation within forebrain principal neurons did not alter anxiety-like responses. A point for consideration is that the dose of CNO (0.5 mg/kg) used in our study may not activate Gi signalling as effectively as it influences Gq signalling, given differences in affinity of CNO for hM4Di versus hM3Dq DREADDs (Gomez et al., 2017). However, it is important to keep in mind that doses of CNO as low as 0.3 mg/kg have been used to successfully inhibit specific neuronal populations expressing hM4Di (Krashes et al., 2011), and the decline noted in c-Fos within the hippocampus in our results indicates inhibition at the dose used in our study. However, further experiments across a wider dose range of CNO would be required prior to concluding that hM4Di-mediated DREADD inhibition of CamKIIα-positive forebrain excitatory neurons does not influence anxiety-like behaviour. We did note a difference in baseline total locomotion in CamKIIα-tTA:TetO-hM4Di bigenic mice as compared to the CamKIIα-tTA:TetO-hM3Dq bigenic cohort. While both the bigenic strains share a similar genetic background, they do appear to exhibit distinct baseline locomotor activity when placed in novel arenas, which could result from genetic changes at the level of quantitative trait loci across several generations of breeding these distinct bigenic mouse lines. Thus, a direct comparison of locomotor activity across these two bigenic lines needs to factor in these baseline differences, and we have restricted our statistical comparisons to the effect of acute CNO-treatment with corresponding vehicle-treated controls within the same bigenic line. The DREADD ligand CNO, used in our study, is known to metabolise into clozapine and *N-*desmethylclozapine (Manvich et al., 2018), that target endogenous receptors (Meltzer, 1994; Lameh et al., 2007). Studies have reported behavioural effects of CNO administration in the absence of DREADD expression (MacLaren et al., 2016), possibly via the metabolites of CNO (Manvich et al., 2018). Administering CNO to the C57BL/6J mice, the background strain, did not alter anxiety or despair-like behaviour, providing support to the evidence that the anxiolytic behavioural effects noted upon CNO administration to CamKIIα-tTA:TetO-hM3Dq mice are specific to hM3Dq DREADD-mediated activation of forebrain excitatory neurons.

Previous studies have capitalised on optogenetic and chemogenetic tools to delineate the contribution of specific circuits, including the ventral hippocampus (vHPC), mPFC, BNST, and amygdala (Nieh et al., 2013; Adhikari, 2014; Burnett and Krashes, 2016) in the regulation of anxiety-like behaviours in rodent models. Strategies that result in broader activation patterns likely encompassing multiple circuits simultaneously, and their impact on these anxiety-related behavioural assays have not been extensively explored. We have used a CamKIIα-driven transgene system, which successfully drives the expression of the relevant DREADD in the hippocampus and cortex, albeit with higher binding reported in the hippocampus (Alexander et al., 2009). The cortex and hippocampus are highly implicated in regulating emotional behaviour given their reciprocal connections with other limbic brain regions (Rajmohan and Mohandas, 2007), and control over the stress response pathways (Jankord and Herman, 2008). Although the circuits involved in the modulation of anxiety-like behaviour are distributed in a brain-wide network, the role of a few specific circuits is better delineated. The mPFC and vHPC along with the amygdala form the emotional triad that integrate sensory information, contextual information, and valence (Adhikari, 2014; Fastenrath et al., 2014; Reznikov et al., 2016; Hiser and Koenigs, 2018; Willinger et al., 2019); thereby play a key role in regulating various aspects of anxiety-like behaviour. vHPC-mPFC projections have been shown to be necessary for anxiety-related neuronal activity and behavioural outcomes (Adhikari et al., 2010, 2011). Single units in the mPFC preferentially fire in either the open or closed arm of the EPM and are influenced by activity in the vHPC (Adhikari et al., 2011). Both optogenetic and chemogenetic activation of CamKIIα-positive mPFC neurons using viral strategies has been shown to decrease anxiety-like behaviour (Wang et al., 2015; Pati et al., 2018), which is consistent with our result of decline in anxiety-like behaviour following broad activation of CamKIIα-positive forebrain excitatory neurons using the CamKIIα-tTA:TetO-hM3Dq bigenic mouse line. Interestingly, optogenetic activation of the granule cells in the DG reduces anxiety-like behavioural responses, and increases exploratory behaviour on approach-avoidance conflict based tasks (Kheirbek et al., 2013). The CamKIIα-tTA:TetO-hM3Dq bigenic mouse line used in our study, would likely recruit both the hippocampus and mPFC, and it is not possible to parse out the relative contribution of these individual forebrain circuits to the anxiolytic behaviours observed following CamKIIα-positive forebrain excitatory neuron activation. Rather, our results inform us that such broad activation of forebrain principal neurons is associated with a robust decrease in anxiety-like behaviour, possibly through the recruitment of both the hippocampus and the mPFC, each of which have been individually shown to influence anxiety-like responses (Calhoon and Tye, 2015; Tovote et al., 2015). Further studies are required to address the hierarchy of contribution of individual forebrain circuits under ethological contexts, wherein more than one individual circuit that modulates anxiety-like behavioural responses is recruited simultaneously.

Recent clinical studies using deep brain stimulation (DBS) of specific limbic brain regions have demonstrated powerful symptomatic relief through an influence on the regulation of negative emotional states and affect, likely via the recruitment of multiple networks implicated in the neurocircuitry of psychopathology (Velasques et al., 2014). Preclinical studies serve to clarify the impact of DBS delivered to specific brain regions, factoring in the nature of frequency stimulation, recruitment of specific interconnected networks, and the associated consequences on a variety of mood-related behaviours (Reznikov et al., 2016). Interestingly, DBS stimulation to the infralimbic prefrontal cortex reduced anxiety-like behaviour in a preclinical post-traumatic stress disorder (PTSD) model (Reznikov et al., 2018). High frequency stimulation to the hippocampus is reported to enhance fear extinction learning and reduce fear recall (Farinelli et al., 2006; Deschaux et al., 2011). Optogenetic excitation of ventral DG granule cells evokes an anxiolytic response, whereas ventral DG granule cell inhibition has no effect on anxiety-like behaviour (Kheirbek et al., 2013). However, in the context of the hippocampus studies using both optogenetic and electrical stimulation indicate differing results on fear extinction/recall and anxiety-like behaviour, based on the temporal context, hippocampal subfield targetted, the nature of stimulation used and the behavioural paradigm (Garcia et al., 2008; Deschaux et al., 2011; Goshen et al., 2011; Lovett-Barron et al., 2014). Our results indicating that acute chemogenetic activation of forebrain principal neurons can elicit an anxiolytic state in diverse behavioural tasks, suggesting that these behavioural consequences may arise through recruitment of the hippocampus and interconnected neocortical circuits, thus influencing state-dependent anxiety in ethologically relevant behavioural paradigms.

## Data Availability Statement

The raw data supporting the conclusions of this manuscript will be made available by the authors, without undue reservation, to any qualified researcher.

## Ethics Statement

All procedures involving animal handling and treatment were carried out in accordance with the guidelines of the Committee for the Purpose of Control and Supervision of Experiments on Animals (CPCSEA), Government of India and were approved by the TIFR Institutional Animal Ethics Committee.

## Author Contributions

SP, SS, and VV designed the experiments and wrote the manuscript. SS and PC performed the Western blotting experiments. SS, SP, PT, and TB performed the behavioural experiments and data analysis. SS performed the immunohistochemical analysis.

## Conflict of Interest

The authors declare that the research was conducted in the absence of any commercial or financial relationships that could be construed as a potential conflict of interest.
